# Feasibility of a wearable self-management application for patients with COPD at home: a pilot study

**DOI:** 10.1186/s12911-024-02461-y

**Published:** 2024-03-05

**Authors:** Robert Wu, Eyal de Lara, Daniyal Liaqat, Salaar Liaqat, Jun Lin Chen, Tanya Son, Andrea S. Gershon

**Affiliations:** 1 University Health Network, Toronto, Canada; 2 University of Toronto, Toronto, Canada; 3 Sunnybrook Health Sciences Center, Toronto, Canada

**Keywords:** COPD, Wearable, Smartphone, Self-management, Smartwatch, Remote monitoring

## Abstract

**Background:**

Among people with COPD, smartphone and wearable technology may provide an effective method to improve care at home by supporting, encouraging, and sustaining self-management. The current study was conducted to determine if patients with COPD will use a dedicated smartphone and smartwatch app to help manage their COPD and to determine the effects on their self-management.

**Methods:**

We developed a COPD self-management application for smartphones and smartwatches. Participants were provided with the app on a smartphone and a smartwatch, as well as a cellular data plan and followed for 6 months. We measured usage of the different smartphone app functions. For the primary outcome, we examined the change in self-management from baseline to the end of follow up. Secondary outcomes include changes in self-efficacy, quality of life, and COPD disease control.

**Results:**

Thirty-four patients were enrolled and followed. Mean age was 69.8 years, and half of the participants were women. The most used functions were recording steps through the smartwatch, entering a daily symptom questionnaire, checking oxygen saturation, and performing breathing exercises. There was no significant difference in the primary outcome of change in self-management after use of the app or in overall total scores of health-related quality of life, disease control or self-efficacy.

**Conclusion:**

We found older patients with COPD would engage with a COPD smartphone and smartwatch application, but this did not result in improved self-management. More research is needed to determine if a smartphone and smartwatch application can improve self-management in people with COPD.

**Trial registration:**

ClinicalTrials.Gov NCT03857061, First Posted February 27, 2019.

**Supplementary Information:**

The online version contains supplementary material available at 10.1186/s12911-024-02461-y.

## Background information 

There are 88,000 hospitalizations for COPD exacerbations in Canada annually with a readmission rate of 19–24% at 30 days and 35–43% at 90 days [1–4]. The cost of a COPD hospitalization is on average $10,000 [5]. Patients hospitalized for COPD experience reduced quality of life, decreased physical activity, diminished mental health, increased risk of future hospitalization, and increased risk of death [6]. People who have been hospitalized with COPD are physically and psychologically weakened [7]. Despite this, they are tasked with both physical and mental challenges to maintain their health. This may leave them feeling overwhelmed and unable to cope. Once back at home, they strive to adhere to advice but often feel discouraged. They often struggle to readjust to what they can and can no longer do. They struggle to regain a sense of control [7].

Mobile technology such as smartphones may provide an effective method to improve care at home by supporting, encouraging, and sustaining self-management among people with COPD. Self-management interventions delivered through mobile technology can provide information and instruction while facilitating goal setting and self-monitoring [8]. Previous interventions have consisted of educational and motivational content related to smoking cessation, exercise, diet and symptom management. A Cochrane review of mobile technology for self-management in COPD found three randomized controlled trials with a total of 557 participants with COPD. All three studies included activity goal setting. Two interventions included pedometers and the third involved computer-tailored behavioral support. This review found that interventions delivered via smart technology significantly improved health related quality of life and activity levels [9]. Of note, the one trial that did not involve wearables showed no difference in quality of life [10]. However, the review found that evidence was of poor quality and further research is required. Of note, this systematic review also found that there were no changes in hospitalizations and that it was difficult to sustain participant engagement after 4 months.

Wearable technology such as smartwatches is a unique form of mobile technology that may increase self-management and engagement by providing self-monitoring through continuous collection of passive information such as activity and vital signs. We hypothesized that a smartphone and smartwatch application may improve self-management in patients with COPD through increased education about and engagement in their condition. We developed a smartphone and smartwatch app to support people managing their COPD. We conducted a pilot study to determine the feasibility of using a dedicated smartphone and smartwatch app in people with COPD. Specifically, we aimed to determine if they would use the app and, if they did, would it improve self-management of their COPD.

## Methods

The current study was conducted to determine if patients with COPD will use a dedicated smartphone and smartwatch app to help manage their COPD and, if so, to determine the effects on their self-management.

### Study design

Prospective before-after cohort feasibility study.

### Study setting

Toronto and Thunder Bay, Ontario, Canada. The study was approved by the UHN Research Ethics Board and registered as a clinical trial (ClinicalTrials.Gov NCT03857061).

### Study population

Through posters within hospitals and respirology clinics, we recruited patients who had COPD confirmed by pulmonary function testing, were age 40 and older, were able to speak English and who had had an exacerbation of their COPD in the previous 12 months. Patients who had an exacerbation of their COPD were studied as it was believed they would be the ones to derive greatest benefit from the intervention. People with other pulmonary conditions such as bronchiectasis or asthma, who had a life expectancy of less than 6 months and/or who had another medical condition that would impair their ability to use a smartwatch and smartphone, such as dementia, were excluded. As this was a feasibility study, a convenience sample size of approximately 30 was chosen as a balance of providing sufficient information on feasibility but also having a high probability of attaining enrollment.

### Study intervention

Participants were provided with a Samsung smartphone (Note 9) and a smartwatch (Galaxy Watch) with a cellular data plan. Our self-management intervention consisted of an application developed based on patient feedback on what COPD patients felt would be helpful when managing their disease [11]. We interviewed four people with lived experience of COPDs in the process of developing the app by asking to them to use the different functions beta versions and provide feedback. Modifications were then made to improve functionality and usability. Specifically, patients requested ability to track their symptoms as well as their activity and oxygen saturation. They also requested education to help manage their symptoms. The application provided functionality to monitor and display heart rate, oxygen saturation, activity, and coughing. Heart rate and activity were passively measured through the smartwatch. Oxygen saturation was actively measured by the patient applying their finger to an optical sensor on the smartphone. Participants could enter and review data about their coughing. Information was displayed on a user interface designed to engage with the participants, provide feedback on their sensor data, and aid in self-management. Other app features included educational COPD resources including pursed lip breathing video exercises, a COPD Action plan in which patients could enter their plan of action in case of worsening symptoms, and medication reminders. There were motivational messages encouraging participation. For activity, participants could set their goal of number of steps per day and would receive positive feedback for exceeding their goal (Eg. “Well done, you’ve beat your activity goal by 15 min”). (Supplementary Figures S1-4 for app screenshots) Participants were trained how to use the smartphone app and the smartwatch during the initial in-person onboarding session by research personnel with training that typically took less than one hour.

### Outcomes

Participants were followed for 6 months. To determine if people used the app, we measured usage of the app in general and in the different smartphone app functions described above. Measures included use of oxygen saturation, completing the daily symptom questionnaire, and performing breathing exercises. To determine if the app changed self-management, the primary outcome was a change in the Mastery sub-section of the Chronic Respiratory Disease Questionnaire at 6 months. Secondary outcomes included changes in the following:

1. Quality of life as measured by the Chronic Respiratory Disease Questionnaire (CRQ), a 20 item with 7-point Likert scale with four sub-sections: dyspnoea, fatigue, emotional function and mastery. Higher scores correspond to higher health related quality of life [12, 13].
2. Clinical COPD disease control as measured by the Clinical COPD Questionnaire (CCQ) [14]. The CCQ consists of 10 items with scores ranging from 0 to 6 with a higher score indicating worse disease control. There are three subscales: symptom, functional state and mental state.
3. Self-efficacy as measured by the COPD Self-Efficacy Scale (CSES) which consists of 34 items comprising of five subscales describing negative affect, intense emotional arousal, physical exertion, weather/environment and behavioral risk factors. Each scale ranges from 1 to 5 with higher scores indicating better self-efficacy [15].
4. Health Related Quality of Life using the St George’s Respiratory Questionnaire (SGRQ) [16]. The SGRQ has 17 items that has three domains: symptoms, activity and impacts (psychosocial), as well as a total score. Scores range from 0 to 100, and higher score indicating worse health status.
5. Symptom scale with the MRC Dyspnea Scale, a 5-point scale describing breathlessness with higher scores indicating worsening breathlessness.

We also asked participants to complete a COPD symptom survey daily through the smartphone app to determine when an exacerbation occurred [17]. The daily symptom score was used to define COPD exacerbations in accordance with previous definitions [17]. The score was a simple sum of major symptoms (dyspnea, sputum purulence, and sputum volume) each worth 5 points and minor symptoms (nasal congestion, wheeze, sore throat and cough) each worth 1 point. The beginning of a COPD exacerbations was defined as a score of 6 or greater for 2 consecutive days and ended with a score of 0 for 5 days. The number of exacerbations during study participation was of interest to determine the feasibility of a larger study.

### Data collection

Baseline demographics, disease characteristics, comorbidities and digital literacy data were collected upon enrollment. To determine digital literacy, participants were asked about technology use, technology in the home, self-reported technical efficacy and highest education obtained. Usage of the different smartphone app functions was measured through the app. Questionnaires to capture primary and secondary outcomes were administered through surveys at enrollment and participant offboarding.

### Data analysis

Descriptive statistics were used to describe the cohort demographics, disease characteristics, and comorbidities as well as app usage data. Changes in questionnaires scores were tested using paired non-parametric analyses. Statistical analyses were conducted using Python. *P* values of ≤ 0.05 were considered significant.

## Results

### Participants

From February 2019 to November 2020, 34 patients were followed for an average of 161 days (SD 103, median 166 days, IQR 115–191). Six participants dropped out within the first 30 days.(Figure S5) Mean age was 69.8 years, and half of the participants were women. Participants had been diagnosed with COPD for an average of 9.3 years, and 32% used home oxygen. In terms of technology use, approximately only two thirds reported using a smartphone before, and one third had used a smartwatch or wearable previously. Most (88%) had internet at home (Table 1).

**Table 1:** Patient characteristics

**Characteristic**		*n* = 34
Age – Mean (SD)		69.8 (9.1)
Women n (%)		17 (50%)
Ethnicity n (%)	Asian	2 (5.9%)
	Black	1 (2.9%)
	White	30 (88.2%)
	Indigenous	1 (2.9%)
Body mass index (SD)		27.4 (5.8)
Charlson Comorbidity Index Score n (%)	2	5 (14.7%)
	3	7 (20.6%)
	4	12 (35.3%)
	5	7 (20.6%)
	6	3 (8.8%)
**COPD related characteristics**		
Years of COPD (SD)		9.3 (5.6)
Current smoker (in the last 3 months), n (%)		8 (23.5%)
Current or prior smoker, n (%)		30 (88.2%)
Home oxygen, n (%)		11 (32.4%)
Has a COPD action plan, n (%)		15 (44.1%)
COPD medication, n (%)	Short-acting beta agonist	22 (71.0%)
	Long-acting beta agonist	20 (64.5%)
	Short-acting anticholinergic	5 (16.1%)
	Long-acting anticholinergic	19 (61.3%)
	Inhaled corticosteroid	21 (67.7%)
FEV1 (SD)		55.8% (20%)
FVC (SD)		70.8% (20%)
Average number of acute exacerbations of COPD in the last year requiring antibiotics or oral steroids but not requiring ED or hospitalization n (SD)		1.6 (2.5)
Average number of hospitalizations for COPD, flu or pneumonia in the last year n (SD)		0.6 (1.8)
Health status (self described) n (%)	Very poor	1 (3%)
	Poor	5 (15.2%)
	Fair	15 (45.5%)
	Good	9 (27.3%)
	Very good	3 (9.1%)
Medical Research Council Dyspnea Scale n (%)	Not troubled by breathlessness, except with strenuous exercise	5 (15.2%)
	Troubled by shortness of breath when hurrying on the level or walking up a slight hill	7 (21.2%)
	Walks slower than people of the same age on the level, stops after a mile or so, or stops after 15 min walking at own pace	15 (45.5%)
	Stops for breath after walking about 100 yds or after a few minutes on level ground	6 (18.2%)
	Too breathless to leave the house or breathless when dressing or undressing	0
**Digital literacy**		
Smartphone use—current or ever		22 (64.7%)
Smartwatch/wearable current or ever		12 (35.3%)
WIFI at home		30 (88.2%)
Use a computer at home (desktop, tablet, laptop)		30 (88.2%)
Technical Efficacy (self-reported) n (%)	High	11 (33.3%)
	Medium	18 (54.5%)
	Low	4 (12.1%)
Highest Education obtained (partial or complete) n (%)	High school	5 (15.2%)
	College or University	21 (63.6%)
	Postgraduate	7 (21.2%)

*COPD* Chronic obstructive pulmonary disease, *SD* standard deviation, *FEV1* Forced Expiratory Volume in 1 s, *FVC* Forced Vital Capacity

### App use

Some participants used the app sporadically, whereas there were active users who used the app at least 50% of days enrolled (Table 2). Active users used the app 2.2 times per day (SD 2.4) in the first month, which declined to 1.0 times per day (SD 1.1) by month 6. They spent 8.5 min per day (SD 9.0) in the first month, but this declined to 3.2 min per day (SD 3.0) by month six. Usage varied by component (Table 2). Recording steps through the smartwatch, completing the daily symptom questionnaire, checking oxygen saturation, and performing breathing exercises were the most used functions.

**Table 2:** Application usage by month

	Month					
	1	2	3	4	5	6
All users (n)	28	27	27	25	23	22
Active Users—used the app at least 50% of days enrolled (n)	28	21	17	15	15	14
App usage in minutes / day (SD)	8.5 (9.0)	5.1 (6.0)	4.4 (4.7)	4.0 (4.2)	3.7 (3.9)	3.2 (3.0)
App uses / day (SD)	2.2 (2.4)	1.6 (2.2)	1.5 (2.0)	1.3 (1.5)	1.2 (1.2)	1.0 (1.1)
Recorded steps through smartwatch	79%	73%	67%	75%	76%	66%
Entered ‘Daily Symptom’ questionnaire	72%	66%	61%	66%	66%	64%
Checked oxygen saturation on phone	69%	50%	35%	38%	45%	41%
Performed breathing exercises on phone	58%	42%	28%	29%	35%	33%
Recorded taking their medications	52%	39%	25%	22%	28%	24%
Viewed action plan	21%	10%	7%	9%	8%	5%
Recorded exercise	18%	5%	5%	6%	5%	5%
Recorded coughing description	17%	12%	7%	9%	8%	6%

With respect to physical activity, the median number of days when steps were recorded was 141 days (IQR 103–164), or 73% of days that participants were enrolled. On average, participants took 1,847 steps per day (SD 1,378). Twenty-six patients had over 30 days of activity data recorded. Among this group, daily steps decreased by 3.3 steps per day (SD 9.6 steps per day).

### Self-management, quality of life, symptoms and outcomes

Baseline CRQ, SGRQ were consistent with moderately limited health status. Similarly, there was moderate disease control (CCQ) and self-efficacy (CSES) at baseline. Participants did not have a significant change in the Mastery sub-section of the Chronic Respiratory Disease Questionnaire after using the app for 6 months (Table 3). Likewise, there was also no significant change in overall total scores of health-related quality of life (SGRQ, CRQ), disease control (CCQ), self-efficacy (CSES) or in dyspnea symptoms (MRC). The only significant change was a reduction in self-efficacy on the Emotional Arousal Subscale of the CSES.

**Table 3:** Changes in self-management, quality of life and self-efficacy scales

Scale	N^a^	Baseline Mean	SD	End Mean	SD	*P* value
CRQ Dyspnea Subscale	27	3.7	1.3	3.9	1.2	.88
CRQ Fatigue Subscale	27	3.8	1.4	4.0	1.2	.38
CRQ Emotional Function Subscale	27	4.8	1.3	4.8	1.3	.97
CRQ Mastery Subscale	26	5.0	1.2	5.1	1.1	.57
CCQ Symptom	25	2.4	1.1	2.3	1.1	.30
CCQ Functional state	25	2.3	1.0	2.3	1.3	.55
CCQ Mental state	25	2.0	1.6	2.2	1.4	.73
**CCQ Mean**	25	2.3	1.0	2.2	1.1	.38
CSES Negative Affect Subscale	24	3.7	0.8	3.3	0.8	.051
CSES Emotional Arousal Subscale	24	3.7	0.7	3.3	0.7	.008
CSES Physical Exertion Subscale	24	2.8	1.0	2.7	1.0	.42
CSES Weather/Environment Subscale	24	3.0	0.9	3.0	0.8	.99
CSES Behavior Subscale	24	3.5	0.9	3.2	0.9	.13
**CSES Total**	24	3.4	0.8	3.1	0.7	.09
SGRQ Symptoms Subscale	25	50.1	20.5	49.4	18.3	.85
SGRQ Activity Subscale	25	70.4	17.9	70.9	20.1	.85
SGRQ Impacts Subscale	25	34.1	15.2	35.6	18.8	.98
**SGRQ Total**	25	47.8	13.7	48.6	15.5	.96
**MRC Dyspnea Scale**	25	2.7	0.96	2.6	0.97	.40

*CRQ* Chronic Respiratory disease Questionnaire dimensions range from 1 (worst health) to 7 (best health)

*CCQ* Clinical COPD Questionnaire dimensions range from 6 (worst) to 0 (best) disease control

*CSES* COPD Self-Efficacy Scale dimensions range from 1 (worst) to 5 (best) confidence in managing and controlling dyspnea

*SGRQ* St George’s Respiratory Questionnaire dimensions range from 100 (worst health) to 0 (best health)

*MRC Dyspnea Scale* Medical Research Council grade of dyspnea with dimensions range from 5 (worse) to 1 (least) dyspnea symptoms

^a^Considered only those who completed at least 1 month of follow up (*n* = 28) and completed the specific questionnaire

During this time, 3 of 34 patients reported a total of 7 COPD hospitalizations due to exacerbations. In addition, as revealed by participants’ symptom questionnaire, there were a total of 20 exacerbations with a median length of 11 days.

## Discussion

We evaluated a smartphone and smartwatch application designed to improve self-management of people with COPD. We found patients would engage with the smartphone and smartwatch application despite an average age of 69 years. We found no significant difference in self-management with use of the app. There was a statistically significant worsening in the Emotional Arousal subscale of the COPD self-efficacy scale.

We found that some people with COPD will interact with a mobile app and will use a wearable. Our participants had step data for approximately 75% of possible days enrolled. Similarly, daily symptom surveys were entered approximately 66% of the time. On average, participants regularly checked their oxygen saturation every other day and also performed breathing exercises every three days through the app.

There are likely several reasons why our smartphone and smartwatch application did not show a significant difference in self-management. Firstly, *health literacy* and *patient activation* are increasingly recognized elements of self-management interventions[18]. Improving health literacy helps patients develop a skillset to better manage their health. Patient activation helps patients develop a mindset to help them change their lifestyle behaviors. While the app was designed to increase healthy behaviors by increasing monitoring of activity, coughing and heart rate and by providing exercises and encouragements, the intervention was passive and may not have addressed health literacy or patient activation sufficiently. Participants were instructed how to use it initially, but little reinforcement for ongoing use was provided. The intervention could be strengthened by incorporating more robust regular interactions known to increase patient activation such as health coaching and motivational interviewing [19]. Other trials of self-management have also incorporated more robust regular interactions such as monthly calls [20]. Incorporating a virtual pulmonary rehabilitation program would be another way to increase both health literacy and patient activation. Participants can be further engaged to use the app by improving the interaction with their primary care physician and respirologists. Engaging their care team to reinforce messages of the importance of physical activity or smoking cessation could increase overall effectiveness of self-management.

The literature is not clear whether mobile applications such as smartphones and/or smartwatches improve the outcomes in patients with COPD. A recent systematic review found inconsistent results in influencing exacerbations, quality of life, physical activity and fatigue [21]. Interventions studied in the systematic review included the use of pulse oximeters and activity monitors. Patients were often asked to answer daily questions, and there was increased contact and monitoring provided. Education was provided, goal setting was encouraged and there were reminders to do breathing exercises [21]. Comparing to other domains such as heart failure, diabetes and asthma, digital health interventions in COPD appear to be less effective overall [22]. While digital tools may have limited effectiveness in isolation, these tools may be effective as part of an larger intervention that includes pulmonary rehabilitation, increasing monitoring, and enhanced communication with the health care team.

The Emotional Arousal subscale of the COPD Self-Efficacy Scale worsened with use of the app [15]. This instrument asks participants to rate their confidence in managing breathing difficulty in specific situations. While other COPD Self-Efficacy subscales focus on weather (e.g. humidity) or physical exertion (e.g. lifting heavy objects), the Emotional Arousal subscale focuses on their ability to manage when feeling emotions such as being excited, upset, angry or tired. This statistically significant difference may have been due to multiple statistical comparisons. If there indeed was a true difference in the Emotional Arousal subscale, it may have been due to the smartphone app or smartwatch providing false hope about improvement in their ability to manage situations. Alternatively, self-efficacy has been shown to decrease in patients with COPD over time [23], and the observed significant difference may be due to the lack of a control group. Nevertheless, self-efficacy would be important to monitor in future studies to ensure there are no unintended consequences.

A major limitation of our study was the lack of a control group. As COPD is a chronic progressive illness, quality of life worsens with duration of illness [24]. Thus people could have had improved self-management, but it was eroded by progression of their disease during the study period. We also did not measure confounding factors such as the use of exercise programs or pulmonary rehabilitation. Participants using the app and wearable were recruited through posters and were likely to be more comfortable with technology and self-motivated than the average person with COPD. It is unclear how representative our sample is of the general COPD population. We did employ two measures of COPD-specific health-related quality of life – the SGRQ and the CRQ. While this could have provided added information such as changes in subscales, it may have contributed to participant fatigue. We examined self-management as the app was designed to help improve self-management, but ultimately, it is an apps’ impact on patient experience, quality of life and clinical outcomes, such as reduced hospitalizations for COPD exacerbations, that need to be investigated. The COVID-19 pandemic impacted this study in multiple ways. First, the pandemic may have reduced COPD exacerbations due to masking and social distancing. Secondly, it also delayed recruitment, particularly during lockdowns. Thirdly, it reduced in-person visits and made follow-up visits much more challenging due to social distancing and lockdowns. Finally, the pandemic may have caused patients’ interest in self-management or participating in research studies to wane.

Future work to create a smartwatch/smartphone app that improves outcomes should address barriers such as low technical efficacy or lower educational training as well as other barriers including language proficiency and socioeconomic status as similar studies ended up recruiting a minority of eligible participants [25, 26]. There is also a strong need to increase the effect of the intervention through interventions such as health coaching, goal setting and clinical team involvement as it appears self-monitoring was not sufficient as a primary component. Instead of asking participants to record their coughing, it may be possible in the future to reduce the participant burden of monitoring through passive monitoring of audio to detect coughing, dyspnea and other features predictive of exacerbations [27]. Further qualitative research would also enhance the important characteristics of a self-management intervention [11, 28]. For example, the infrequent use of the Action Plan feature may be due only needing this infrequently when people are close to an exacerbation. Interviewing participants would help answer this question. Finally, this pilot study was a step to address the knowledge gap in RCTs showing lack of evidence. Once a more effective intervention is developed, further evaluation with an RCT would be necessary.

In summary, we found that older patients with COPD would engage with a smartphone and smartwatch application. Our smartphone and smartwatch application did not show improved self-management in a group of people with COPD. More research is needed to determine if a smartphone and smartwatch application is useful when used as part of a larger program of care for people with COPD to help to improve both self-management and clinical outcomes.

### Supplementary Information

**Supplementary Material 1. **

## Data Availability

The datasets generated and/or analyzed during the current study are not available to share due to limitations of the REB study approval of the initial study protocol.
