# Supplementary material for: Feasibility of a wearable self-management application for patients with COPD at home: a pilot study
Source: BMC Med Inform Decis Mak. 2024 Mar 5;24:66. doi: 10.1186/s12911-024-02461-y (PMC10916068; doi:10.1186/s12911-024-02461-y)
Supplement: Supplementary file 1 — Supplementary Material 1. [file 12911_2024_2461_MOESM1_ESM.pdf]

Supplementary Figures of app screenshots

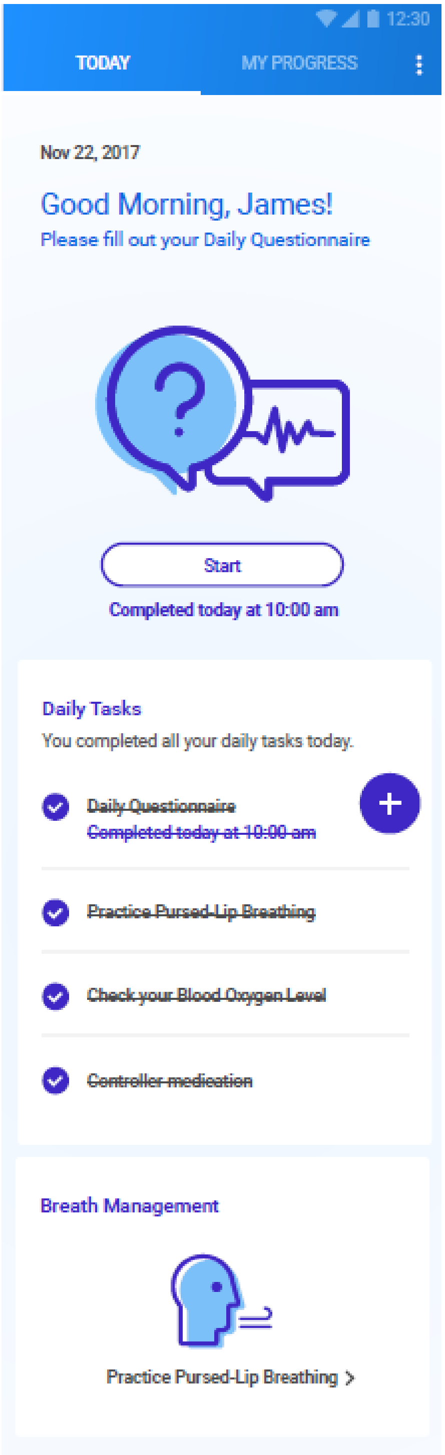

Figure S1: Daily task list

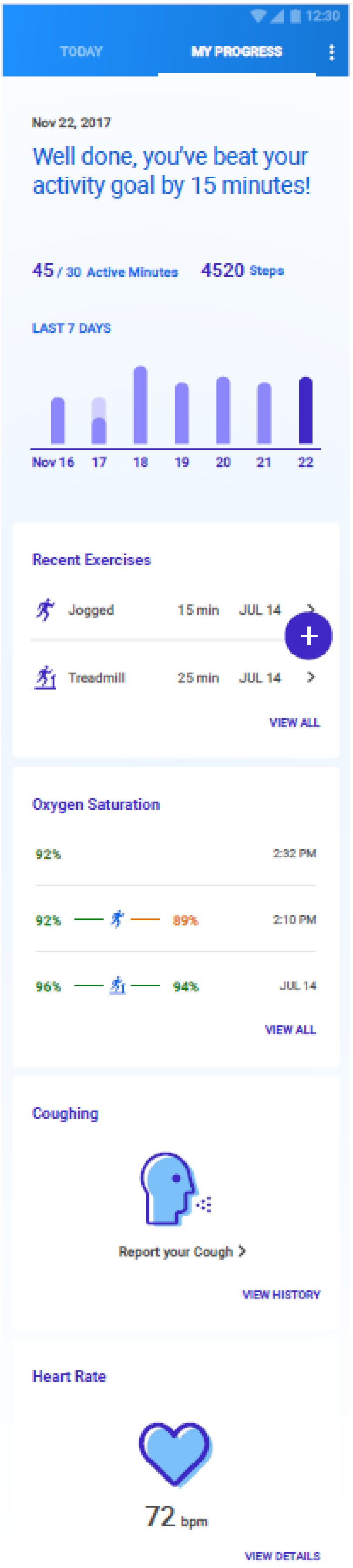
12:30
TODAY
MY PROGRESS
Nov 22, 2017
Well done, you've beat your activity goal by 15 minutes!
45 / 30 Active Minutes
4520 Steps
LAST 7 DAYS
Nov 16
17
18
19
20
21
22
Recent Exercises
Jogged 15 min JUL 14
Treadmill 25 min JUL 14
VIEW ALL
Oxygen Saturation
92% 2:32 PM
92% 89% 2:10 PM
96% 94% JUL 14
VIEW ALL
Coughing
Report your Cough
VIEW HISTORY
Heart Rate
72 bpm
VIEW DETAILS

Figure S2: Reviewing activity data

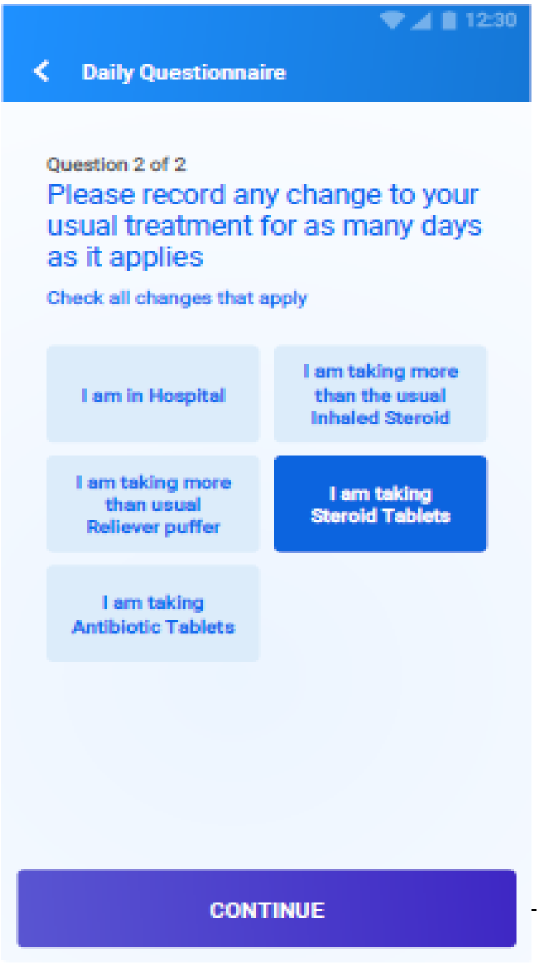

Figure S3: Entering Daily Symptom Questionnaire

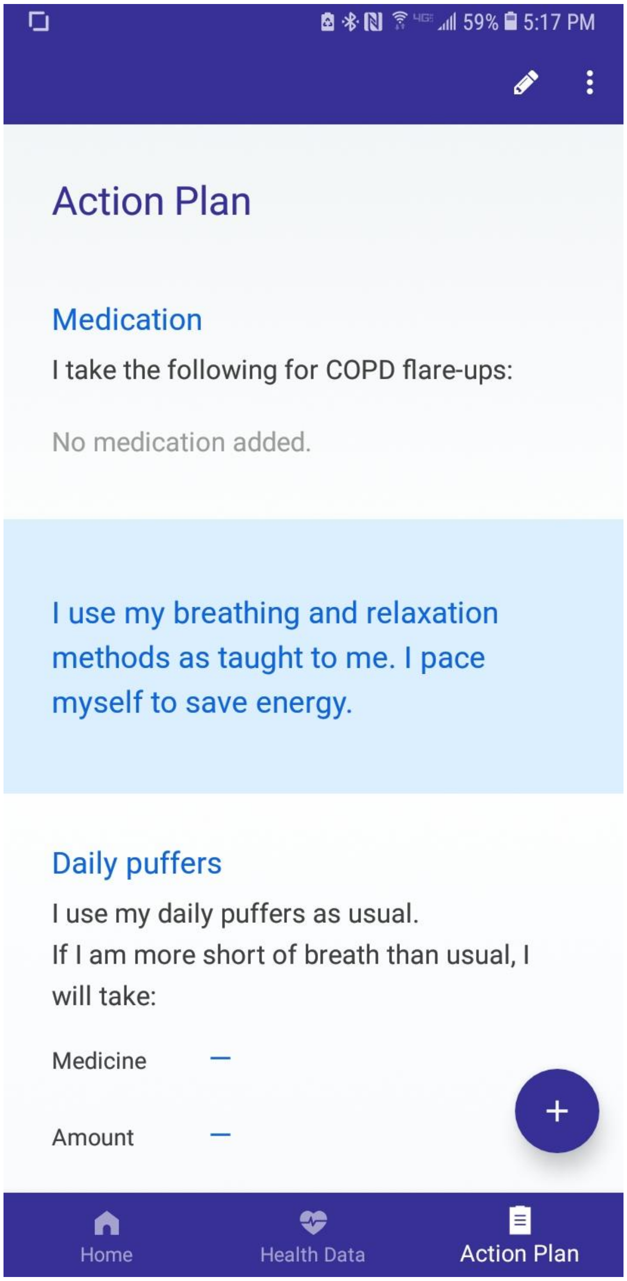

Figure S4: Reviewing Action Plan

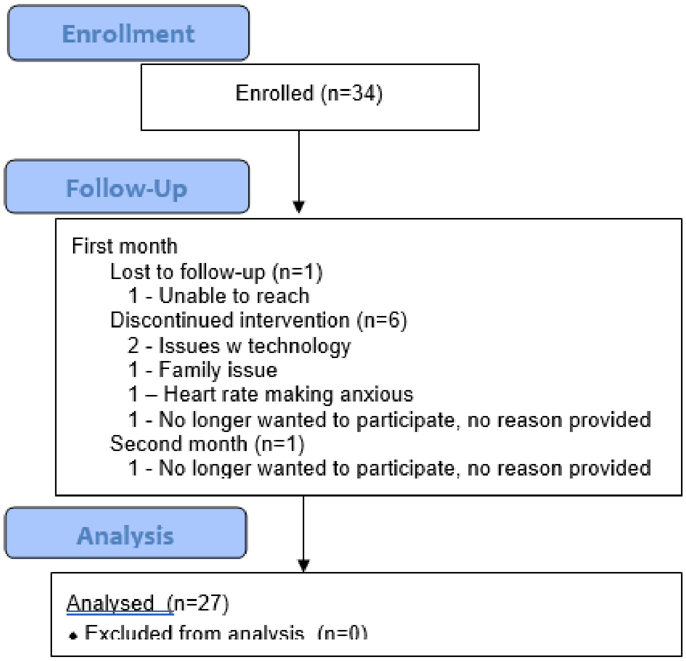

Figure S5: Flow Diagram
